# Antiparasitic effect of synthetic aromathecins on *Leishmania infantum*

**DOI:** 10.1186/s12917-019-2153-9

**Published:** 2019-11-09

**Authors:** Rosa M. Reguera, Raquel Álvarez-Velilla, Bárbara Domínguez-Asenjo, Camino Gutiérrez-Corbo, Rafael Balaña-Fouce, Mark Cushman, Yolanda Pérez-Pertejo

**Affiliations:** 1 Departamento de Ciencias Biomédicas, Universidad de León, Campus de Vegazana s/n; 24071 León (SPAIN), León, Spain; 2 Department of Medicinal Chemistry, and Molecular Pharmacology, College of Pharmacy, and The Purdue Center for Cancer Research, Purdue University, Lafayette, Indiana USA

**Keywords:** *Leishmania*, DNA-topoisomerase IB, Camptothecin, Aromathecins

## Abstract

**Background:**

Canine leishmaniasis is a zoonotic disease caused by *Leishmania infantum*, being the dogs one of the major reservoirs of human visceral leishmaniasis. DNA topology is a consolidated target for drug discovery. In this regard, topoisomerase IB – one of the enzymes controlling DNA topology – has been poisoned by hundreds of compounds that increase DNA fragility and cell death. Aromathecins are novel molecules with a multiheterocyclic ring scaffold that have higher stability than camptothecins.

**Results:**

Aromathecins showed strong activity against both forms of *L. infantum* parasites, free-living promastigotes and intra-macrophagic amastigotes harbored in ex vivo splenic explant cultures obtained from infected BALB/c mice. However, they prevented the relaxation activity of leishmanial topoisomerase IB weakly, which suggests that the inhibition of topoisomerase IB partially explains the antileishmanial effect of these compounds. The effect of aromathecins was also studied against a strain resistant to camptothecin, and results suggested that the trafficking of these compounds is not through the ABCG6 transporter.

**Conclusions:**

Aromathecins are promising novel compounds against canine leishmaniasis that can circumvent potential resistances based on drug efflux pumps.

## Background

Canine leishmaniasis (CanL) is a serious zoonotic disease caused by *L. infantum* in the Old World and *L. infantum chagasi* in the New World. Dogs affected by this disease become reservoirs of human visceral leishmaniasis, being extremely relevant the presence of *L. infantum* as its subspecies in Latin America, mainly in Brazil. Although there are several vaccines in use, and preventive measures including collars impregnated with insecticide or spot-on drops contribute to reduce the endemicity of this disease, the use of drugs is strictly necessary when the signs and symptoms appear in the animal. Nowadays, antimony-based (Sb^V^) drugs, alone or in combination with allopurinol, are considered the gold standard treatment against CanL in southern European countries, whereas the oral drug miltefosine is used as second-line drug [1]. However, the mandatory parenteral administration of Sb^V^ and the multiple side effects of Sb^III^ (product of enzymatic activation of the drug by host enzymes), are some of the causes of treatment interruption, which favors the emergence of relapses in the first-year post-treatment. Related to drug misuse is the emergence of resistant strains that may be triggered by host or parasite factors. Among the factors linked to the host, the most prevalent are those related to the alterations of pharmacokinetic parameters or the immunological system. On the other hand, factors related to parasites include structural modifications of target proteins, along with overexpression of ABC and multiple drug resistance (MDR) proteins [2]. Therefore, drug discovery research in this field is absolutely necessary to find new drugs for the management of CanL.

DNA topoisomerases are consolidated targets for drug development in cancer and infectious diseases. DNA topoisomerase IB (TopIB) is involved in relaxing supercoiled DNA by a DNA breaking and rejoining process. In this process TopIB cleaves one DNA strand by nucleophilic attack from the catalytic tyrosine placed in the active site, which becomes linked to 3′ phosphate end of DNA generating a reversible DNA-enzyme cleavage complex. The unbroken strand rotates through the gap and finally the DNA backbone is rejoined [3]. *Leishmania* TopIB (LTopIB) is interesting from a therapeutic point of view due to its heterodimeric structure, which is dissimilar to the monomeric Top IB found in the rest of animal species [4]. TopIB inhibitors have been classified as TopIB poisons and TopIB inhibitors. TopIB poisons, such as camptothecin (CPT) and other non-CPT compounds like indenoisoquinolines, are anticancer drug leads [5, 6], and several reports have shown their efficacy as trypanocide [7] and leishmanicide drugs [8]. These TopIB poisons trap the cleavage complex preventing the final step of rejoining, intercalating into the DNA-enzyme complex and generating single strand breaks that evolve to double strand breaks when the replication fork collides with the stabilized cleavage complex [3]. On the other hand, TopIB inhibitors do not stabilize the cleavage complex; they inhibit the enzyme, preventing its binding with DNA by the interaction with the enzymatic catalytic domains or with the DNA substrate [3].

**Fig. 1:**
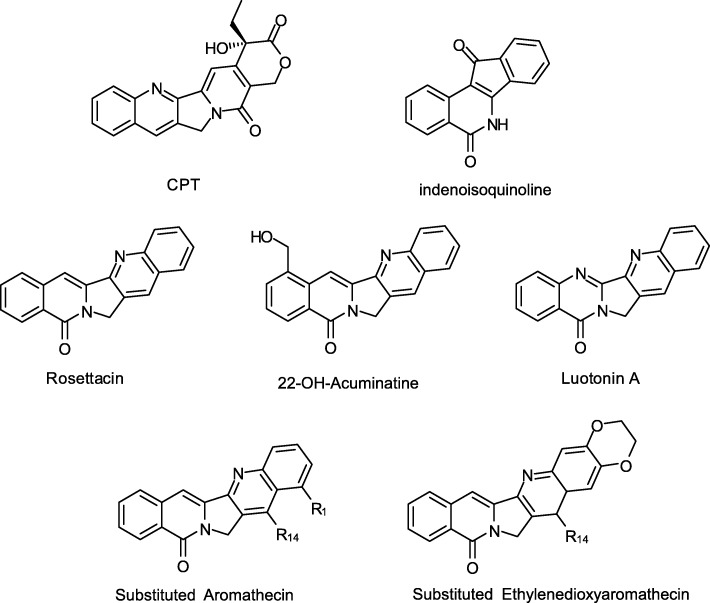
Chemical structure of TopIB poisons. CPT, indenoisoquinoline scaffold, natural aromathecin-like compounds: Rosettacin, Luotonin A and 22-OH-Acumanetin; synthetic aromathecins used in the current work with relevant positions numbered

Aromathecins are a new class of TopI poisons, described as stable hybrids of indenoisoquinolines and camptothecins that also show similarity to the natural product luotonin A, which is a weaker TopIB poison [9] (Fig. 1). Several series of modified and substituted luotonins have been published and some analogues have greater antiproliferative activity than the parent compound. 22-Hydroxyacuminatine [10], a rare natural product, contains the 12H-5,11a-diazadibenzo [b, h] fluoren-11-one system, which is known as “rosettacin” (Fig. 1). Rosettacin derivatives generate aromathecins, which are more stable than camptothecins and maintain lower, but mensurable, topoisomerase poisoning activity [11] (Fig. 1). Further structure-activity studies revealed some trends about these compounds, such as substitutions at C-14 that have been related to an increased anti-cancer activity [11–13] or the ethylenedioxy bridge between C-2 and C-3 that has been associated with an enhanced antitrypanosomal activity [14].

The only TopIB inhibitors FDA approved (irinotecan and topotecan) are CPT analogs. These compounds have several limitations as chemical instability of the CPT alpha-hydroxy-lactone ring and loss of efficacy due to the drug efflux-mediated resistance generation [6]. Therefore, it is necessary the development of new non-CPT TopIB inhibitors that overcome these limitations such as aromathecins.

In *Leishmania* have been described two mechanisms of CPT resistance using strains exposed to increasing concentrations of CPT. The first one involves overexpression of the ABCG6 transporter [15]; the other mechanism presumes the amino acid substitutions Gly185Arg and Asp325Glu in the large subunit of the LTopIB enzyme [16]. In mammalian cells the drug efflux pump involved in CPT resistance is the ABCG2 transporter and it has been described that indenoisoquinolines are poor substrates for this transporter besides the multiple drug resistance (MDR)-1 protein [17].

In this report, we describe the antileishmanial activity of two series of aromathecins, which have been kindly provided by Dr. Mark Cushman (Dpt. of Medicinal Chemistry, Purdue University, Indiana, USA), against both stages of *L. infantum*; free-living promastigotes and intra-macrophagic amastigotes present in splenic explants obtained from infected BALB/c mice. In addition, it was assessed their activity as LTopIB inhibitors and their ability to overcome the transporters involved in CPT resistance.

## Results

Two series of aromathecins (Table 1) have been tested against both stages of *L. infantum,* free-living promastigotes and intracellular amastigotes harbored in mouse splenic cells. Experiments were carried out using a genetically modified *L. infantum* strain that constitutively produces the iRFP protein. This makes it possible to evaluate the viability of parasites measuring the fluorescence emitted at 708 nm [18].

**Table 1:**
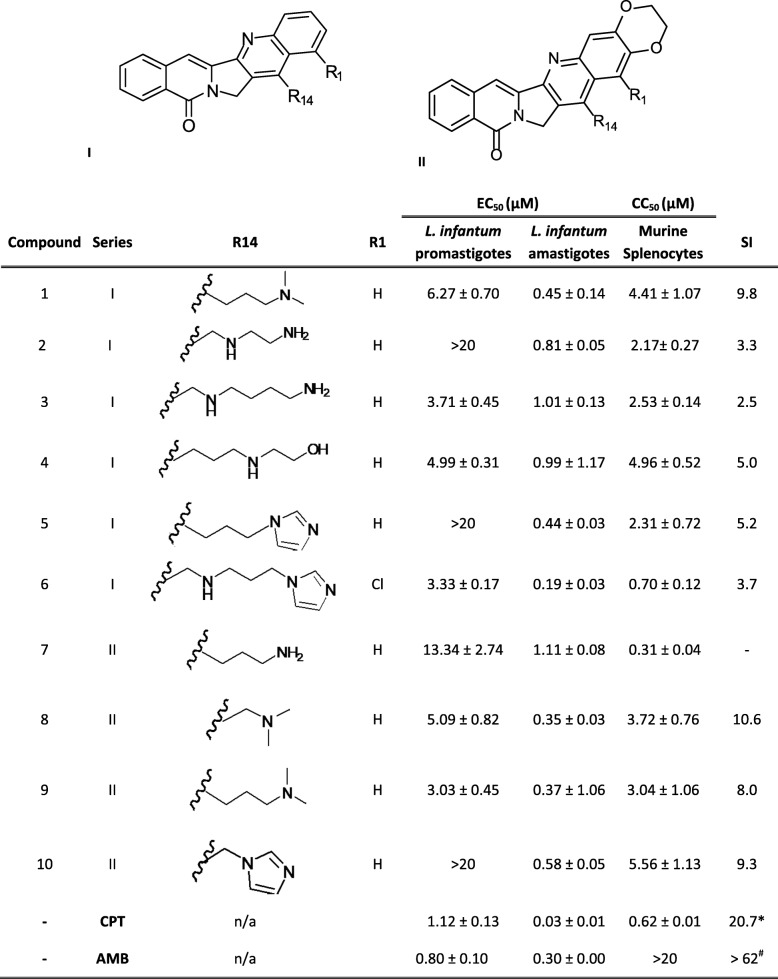
Bioactivity of aromathecines on iRFP-*L. infantum* promastigotes and splenic-infecting amastigotes. Each point represents the average of three different experiments by duplicate

SI: Selectivity Index = CC_50_/EC_50_ (amastigotes)

* See reference [8]

# See reference [19]

In order to enhance the antiproliferative activity and TopIB inhibition, the aromathecins used in this study include different substituents (amines, amino alcohols and nitrogenous heterocycles) at the C-14 position which adding solubility and stability to the DNA-enzyme complex [11] (Table 1). In addition, four of them contain an ethylenedioxy bridge (between C-2 and C-3) in the A-ring (aromathecins **7**,** 8**, **9** and **10**) which is aimed at improving the TopIB inhibitory activity of these compounds [13] (Table 1). Interestingly, all the compounds tested in this work showed lower EC_50_ values in amastigotes than in promastigotes within the micromolar to submicromolar range. Interesting SI values (> 9) were found for compounds **1**, **8** and **10**. The ethylenedioxy bridge does not seem to exert a significant effect on the antileishmanial potency.

**Fig. 2:**
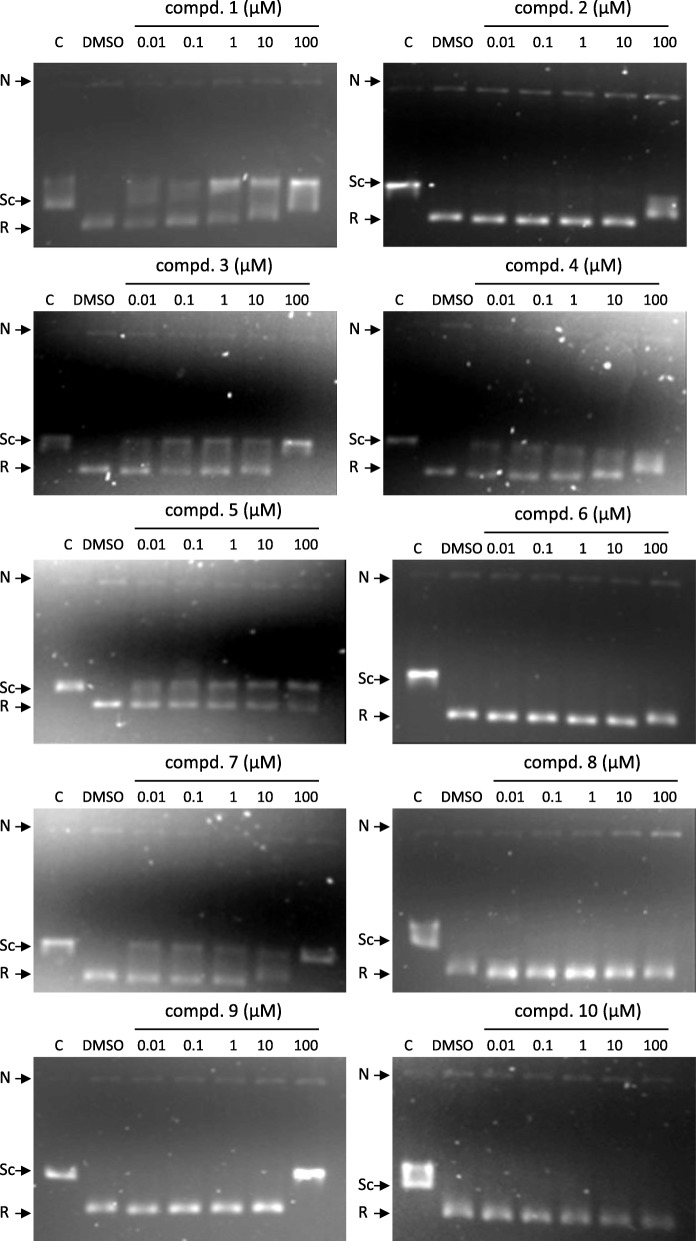
Inhibition of LTopIB relaxation activity of negatively supercoiled pBluescript SK (−) plasmid (pSK) mediated by different aromathecins concentrations (0.01, 0.1, 1, 10 and 100 μM). The control reaction contained 1% DMSO. DNA was separated by gel electrophoresis in 1% agarose containing 0.1 μg/mL ethidium bromide. Gels were visualized with UV illumination. Key: R = relaxed DNA; Sc = supercoiled DNA; N = nicked DNA

In addition to the antileishmanial effect of these compounds, their activity as LTopIB inhibitors was analyzed in vitro (Fig. 2). Recombinant LTopIB was produced in a TopIB-defective yeast platform, and purified by standard chromatographic protocols [20]. The effect of aromathecins (comp. **1** to **10**) on LTopIB was addressed by measuring the relaxation of negatively supercoiled pBluescript-SK DNA plasmid in the presence of different concentrations of these molecules. In order to separate the nicked DNA generated by the cleavage-complex stabilization from the relaxed topoisomers, gels were run in the presence of ethidium bromide. Five compounds (**1**,** 3**,** 4**,** 5** and **7**) showed a partial inhibition of LTopIB, which started at a very low concentration (0.01 μM) and was not concentration dependent. Only a weak band of nicked DNA could be observed with compound **8**, suggesting that these compounds would act as LTopIB inhibitors rather than poisons. The total inhibition of the enzyme was only observed at a high concentration (100 μM) of aromathecins **3**, **7** and **9**; in addition, compounds **6** and **10** did not have any inhibitory effect, indicating that other targets may be involved.

In order to evaluate if aromathecins would share the transporter involved in CPT resistance, one of the major problems related to the loss of effectiveness of CPT derivatives, a CPT-resistant strain was generated. The CPT-resistant strain used was generated by the exposure of *L. infantum* promastigotes to increasing concentrations of CPT, from 0.1 to 20 μM (CPT-20, from now on). CPT-20 showed cross-resistance to other CPT derivatives poisons, such as topotecan (EC_50_ > 100 μM vs EC_50_ = 9.54 ± 0.22 μM in the WT strain) and SN38 (EC_50_ = 40.98 ± 0.3 μM vs EC_50_ = 4.73 ± 0.14 μM in the WT strain). Amplification and sequencing of the LTopIB-encoding genes from CPT-20 and WT strains showed no differences between them. Therefore, it is very likely that the resistance mechanism of CPT-20 is related to the overexpression of the ABCG6 transporter previously described as the drug-efflux pump involved in the CPT resistance mechanism [15]. The CPT-20 strain was susceptible to the aromathecins assayed in this work (**2**,** 5** and **10** were not assayed due to their poor effect against iRFP *L. infantum* promastigotes), showing very similar EC_50_ values to those obtained with iRFP *L. infantum*.

## Discussion

All aromathecins tested have shown leishmanicidal activity with stronger effect over the intracellular amastigote form. Aromathecins have been designed as inhibitors of TopIB enzyme and their mechanism of action would be mediated by the stabilization of the intermediate cleavage-complex [11–13]. However, the results obtained in this work indicate that most of these compounds would act as LTopIB inhibitors rather than LTopIB poisons, due to only with compound **8** we can observe nick DNA from cleavage-complex stabilization.

Unlike the results obtained with *T. brucei* [14], the ethylenedioxy bridge does not seem to exert a significant effect on the antileishmanial potency. Besides, this bridge does not seem to improve the LTopIB inhibitory activity, especially when we compare the activity of compounds **1** and **9**, with the same substitution at C-14 position. The inhibition of LTopIB with compound **9** that has the ethylene bridge only happed at a high concentration whilst compound **1** inhibit the enzyme at very low concentrations.

The leishmanicidal activity of compounds **6** and **10**, which do not have LTopIB inhibitory activity, along with the partial inhibition of the enzyme observed with other compounds indicates that other targets may be involved.

The CPT resistant strain generated in this work by exposition to increased CPT concentrations was susceptible to all the aromathecins tested. These results indicate that aromathecins overcome the ABCG6 transporter, the pump responsible of CPT resistance generation in *Leishmania* [15]. Similar results have been obtained with other non-camptothecin compounds as indenoisoquinolines, which overcome the ABCG2 transporter involved in CPT resistance in mammalian cells, and the multiple drug resistance (MDR)-1 protein [17].

## Conclusions

In conclusion, the aromathecins tested in this work showed a good antileishmanial activity. These results suggest the existence of another mechanism of action complementary to the expected LTopIB poisoning. Structural differences of LTopIB and the mammalian enzyme could explain this behavior. These differences could be used to design new molecules with improved selectivity, whose advantage lies in overcoming the transporter involved in the generation of CPT resistance.

## Methods

### In vitro *L. infantum* promastigotes assay

The antiparasitic activity of the compounds was assessed on the genetically modified iRFP *L. infantum* strain, which constitutively produces the infrared fluorescent protein (iRFP) when cells are viable [18]. iRFP *L. infantum* was grown at 26 °C in M-199 medium (Gibco) supplemented with 25 mM HEPES pH 7.2, 0.1 mM adenine, 0.0005% (w/v) hemin, 2 μg/mL biopterin, 0.0001% (w/v) biotin, 10% (v/v) heat-inactivated fetal bovine serum (FBS) and an antibiotic cocktail comprising 50 U/mL penicillin and 50 μg/mL streptomycin.

### Ex vivo murine splenic explant culture

Primary splenocyte cultures containing intramacrophagic amastigotes were obtained from dissected spleens of female BALB/c mice infected intraperitoneally with 1.5 × 10^9^ metacyclic iRFP *L. infantum* promastigotes, 5 weeks before their sacrifice. Mice were obtained commercially (Janvier-Labs). Animals were housed in specific-pathogen free facilities and they were euthanized by cervical dislocation without anesthesia. These protocols were approved by the Animal Care Committee of University of Leon (project license SAF2017–83575-R), which complies with European Union Legislation (2010/63/UE) and Spanish Act (RD 53/2013).

The spleens were washed with cold phosphate-buffered saline (PBS), cut in small pieces and incubated for 20 min with 5 mL of 2 mg/mL collagenase D (Sigma) prepared in buffer (10 mM HEPES, pH 7.4, 150 mM NaCl, 5 mM KCl, 1 mM MgCl_2_ and 1.8 mM CaCl_2_). After that, the cell suspension obtained was passed through a 100 μm cell strainer, harvested by centrifugation (500×g for 7 min at 4 °C), washed twice with PBS, and cultured at 37 °C under 5% CO_2_ atmosphere in RPMI medium (Gibco) supplemented with 10 mM HEPES, 1 mM sodium pyruvate, 1xRPMI 1640 vitamin mix, 10% (v/v) FBS, 50 U/mL penicillin and 50 μg/mL streptomycin (Calvo-Álvarez et al., 2015).

### Cytotoxicity and selectivity index determination

The viability of cultured parasites in the presence of different concentrations of aromathecins was determined by measuring the reduction of infrared fluorescence emitted by free-living promastigotes/amastigotes with respect to negative controls of each form of the parasite treated with DMSO (up to 0.1% final concentration) as a vehicle. Thus, in order to calculate EC_50_ value, promastigotes or splenic mouse explants, which harbored amastigotes, were incubated with 7 different concentrations of each aromathecin starting from 100 μM and one-third dilutions up to 0.13 μM in duplicate and in three independent experiments. After 72 h incubation at 26 °C, the infrared fluorescence emitted at 708 nm by viable promastigotes/amastigotes was measured in an Odyssey (Li-Cor) infrared imaging system. Similarly, the cytotoxic effect (CC_50_) of aromathecins on uninfected splenic explants from BALB/c mice (cells that naturally harbor the amastigote stage) was measured by the Alamar Blue (Invitrogen) assay. The selectivity index (SI) was determined as the relationship between the CC_50_ value and the EC_50_ value for amastigotes. CC_50_ and EC_50_ were calculated by nonlinear analysis using the Sigma-Plot 10.0 statistical package. The aromathecins were dissolved in DMSO and stored at − 20 °C before their use as fresh aliquots.

### Leishmanial TopIB purification

Expression and purification of LTopIB were carried out according to a previously standardized protocol [20]. LTopIB was purified from yeast strain EKY3 deficient in TopIB activity [*MATα*, *ura*3–52, *his*3Δ200, *leu*2Δ1, *trp1*Δ63, *top1*Δ:*TRP1*], transfected with the pESC-URA plasmid containing both subunits of LTopIB. Cells were grown in yeast synthetic drop-out medium without uracil (Sigma) supplemented with 2% raffinose (w/v) to OD_600_: 0.8–1 and induced for 10 h with 2% galactose (w/v). Yeast were harvested, washed with cold TEEG buffer (50 mM Tris-HCl pH 7.4, 1 mM EDTA, 1 mM EGTA, 10% glycerol) and resuspended to their lysis in 15 mL of 1 x TEEG buffer supplemented with 0.2 M KCl and a protease inhibitors cocktail (Thermo Scientific). Protein extract obtained was loaded on a 5 mL P-11 phosphocellulose column (Whatman International Ltd. England). LTopIB protein was eluted at 4 °C with a discontinuous gradient of KCl (0.2, 0.4, 0.6 M) in TEEG buffer.

### TopIB relaxation activity assay

The effect of aromathecins on recombinant LTopIB was determined by measuring the relaxation of negatively supercoiled pBluescript-SK DNA plasmid (pSK). Thus, 20 μL of reaction mixture (0.5 μg pSK; 10 mM Tris-HCl pH 7.5; 5 mM MgCl_2_; 0.1 mM EDTA; 15 μg/mL bovine serum albumin) along with 0.01, 0.1, 1, 10 and 100 μM of the different aromathecins were incubated at 26 °C during 4 min. The control reaction performed without aromathecins contained 1% DMSO. Reactions were stopped by adding 1% SDS (final concentration), digested with 1 mg/mL proteinase K at 37 °C during 1 h and extracted with phenol/chloroform. DNA was separated by gel electrophoresis in 1% agarose containing 0.1 μg/mL ethidium bromide in 0.1 M Tris borate EDTA buffer (pH 8.0) at 4 V/cm for 16 h. Gels were visualized with UV illumination.

### CPT resistant strain generation

The CPT resistant strain used in this work was generated exposing *L. infantum* promastigotes to increased concentrations of CPT, from 0.1 to the solubility limit of the compound, 20 μM in the case of CPT. Parasites were grown at 26 °C in M199 medium supplemented as described previously, each culture being inoculated at an initial density of 10^6^ cells/mL. The promastigotes cultured in liquid medium in the presence of 20 μM CPT, were plated on semisolid M199 medium containing 20 μM CTP in order to select a single colony.

## Data Availability

The datasets used and/or analysed during the current study are available from the corresponding author on reasonable request.

## Abbreviations

CanL: Canine leishmaniasis
CPT: Camptothecin
iRFP: infraRed Fluorescent Protein
LTopIB: *Leishmania* topoisomerase IB
MDR-1: Multiple Drug Resistance protein 1
SI: Selectivity Index
TopIB: DNA topoisomerases IB
